# Evaluation of self-reported ethnicity in a case-control population: the stroke prevention in young women study

**DOI:** 10.1186/1756-0500-2-260

**Published:** 2009-12-18

**Authors:** Jesse B Mez, John W Cole, Timothy D Howard, Leah R MacClellan, Oscar C Stine, Jeffery R O'Connell, Marcella A Wozniak, Barney J Stern, John D Sorkin, Braxton D Mitchell, Steven J Kittner

**Affiliations:** 1 Department of Neurology, University of Maryland School of Medicine, Baltimore, MD, USA; 2 Medical Research Service, Veterans Affairs Medical Center, Baltimore, MD, USA; 3 Department of Pediatrics, Center for Human Genomics, Wake Forest University School of Medicine, Winston-Salem, NC, USA; 4 Department of Epidemiology and Preventative Medicine, University of Maryland School of Medicine, Baltimore, MD, USA; 5 Department of Medicine, University of Maryland School of Medicine, Baltimore, MD, USA

## Abstract

**Background:**

Population-based association studies are used to identify common susceptibility variants for complex genetic traits. These studies are susceptible to confounding from unknown population substructure. Here we apply a model-based clustering approach to our case-control study of stroke among young women to examine if self-reported ethnicity can serve as a proxy for genetic ancestry.

**Findings:**

A population-based case-control study of stroke among women aged 15-49 identified 361 cases of first ischemic stroke and 401 age-comparable control subjects. Thirty single nucleotide polymorphisms (SNPs) throughout the genome unrelated to stroke risk and with established ancestry-based allele frequency differences were genotyped in all participants. The *Structure *program was used to iteratively evaluate for K = 1 to 5 potential genetic-based subpopulations. Evaluating the population as a whole, the *Structure *output plateaued at K = 2 clusters. 98% of self-reported Caucasians had an estimated probability ≥50% of belonging to Cluster 1, while 94% of self-reported African-Americans had an estimated probability ≥50% of belonging to Cluster 2. Stratifying the participants by self-reported ethnicity and repeating the analyses revealed the presence of two clusters among Caucasians, suggesting that potential substructure may exist.

**Conclusions:**

Among our combined sample of African-American and Caucasian participants there is no large unknown subpopulation and self-reported ethnicity can serve as a proxy for genetic ancestry. Ethnicity-specific analyses indicate that population substructure may exist among the Caucasian participants indicating that further studies are warranted.

## Introduction

Population-based case-control studies are used to identify common susceptibility variants for complex genetic traits; however, population stratification may confound their results [1,2]. Population stratification refers to differences in allele frequencies between cases and controls due to systematic differences in ancestry, rather than association of an allele with disease. To reduce the impact of population stratification, cases and controls are ascertained from the same population and matched on self-reported ethnicity. Some studies indicate that stratifying by self-reported ethnicity (i.e. race) may not adequately adjust for population stratification, specifically in out-bred United States populations [2]. A panel of genetic markers specific to ancestry and unlinked to the disease can be used to evaluate whether self-reported ethnicity can serve as a proxy for genetic ancestry or relatedness [3]. Literature suggests that a panel composed of ~20-40 appropriately chosen markers (SNPs or microsatellites) is sufficient for evaluating a group based ancestry estimation [4], but not individual ancestry estimation. In this report, we genotyped 30 markers selected because of their differing allele frequencies between European Caucasians and Nigerians (Yoruba). We used these markers to determine whether self-reported ethnicity can accurately approximate ancestry in a large biracial population of stroke cases and controls.

## Materials and methods

### Study population

The Stroke Prevention in Young Women (SPYW) Study is a population-based case-control study initiated to examine risk factors for first ischemic stroke in women aged 15-49. All participants were identified from the same population including all of Maryland (except the far Western panhandle), Washington DC, and the southern portions of both Pennsylvania and Delaware. The methods for discharge surveillance, chart abstraction, and case adjudication have been described previously [5]. We determined each subject's case-control status (i.e. determined subjects who had a stroke) blinded to genetic information. Strokes were further classified by subtype according to TOAST (Trial of Org 10172 in Acute Stroke Treatment) [6] including thrombosis or embolism due to atherosclerosis of a large artery (N = 16), embolism of cardiac origin (N = 69), occlusion of a small blood vessel (N = 45), other determined cause (N = 43), undetermined cause (two possible causes, no cause identified, or incomplete investigation) (N = 188). Controls subjects (women without a history of stroke), were identified by random digit dialing and were frequency matched to the cases by age, race, and geographic region of residence. The present analysis includes 762 subjects (361 cases and 401 controls) from this study who self-identified themselves as Caucasian (non-Hispanic) (N = 405) or African-American (N = 357) (see Table 1).

**Table 1:** Characteristics by case-control status

	Case (N = 361)	Control (N = 401)	p-value
Mean age (years)	39.5 ± 0.4	37.8 ± 0.4	0.002

African American (%)	186 (51.5%)	171 (42.6%)	0.003

Hypertension (%)	127 (35.8%)	58 (14.5%)	< 0.0001

Diabetes mellitus (%)	53 (14.9%)	19 (4.8%)	< 0.0001

Current smokers (%)	176 (49.3%)	107 (26.7%)	< 0.0001

Angina-MI (%)	16 (4.5%)	0 (0.0%)	< 0.0001

### SNP selection and genotyping

Twenty ancestry informative markers (i.e. SNPs) were chosen from a HapMap panel previously shown to differ (χ^2 ^> 10) in allele frequencies between individuals from Utah with European ancestry (CEU) and individuals from Nigeria (YRI) [7]. Ten additional SNPs were similarly selected from the Linkage IVb panel (Illumina, San Diego, CA).

Genotyping was conducted using DNA isolated from whole blood using the QIAamp DNA Blood Maxi Kit (Qiagen, Valencia, CA). SNP genotyping was performed by either TaqMan (Applied Biosystems, Foster, CA) or iPLEX (Sequenom, San Diego, CA) methodologies. For each SNP, genotyping for all cases and controls was performed on the same platform.

Following genotyping, four SNPs were excluded from the analyses: three SNPs (rs1021516, rs1648282, rs1011526) exhibited genotype call rates less than 80% and one SNP (rs2695) did not exhibit a difference in allele frequencies between our Caucasian and African-American populations. Hence, 26 SNPs distributed throughout the genome were included in the analyses (Table 2), with 7 of the SNPs genotyped via Taqman and 19 via iPLEX. All SNPs were verified to be unassociated with stroke (additive model) in the total population and stratified by race. All SNPs were verified to be in Hardy-Weinberg equilibrium (χ^2 ^test). Major allele frequency differences between self-reported Caucasians and African-Americans were calculated (χ^2 ^test). Analyses were performed using SAS^®^, Version 9.1 (SAS Institute, Cary, NC) (Tables 1 and 2).

**Table 2:** Twenty-seven SNPs listed by chromosomal location, including genotype call rates, ethnicity-specific allele frequencies, and relative difference between major allele frequency.

Marker/Alleles*	Chromosome	Location	Call rate	Major Allele Frequency-Caucasians	Major Allele Frequency - African Americans	Major Allele Frequency difference between Caucasians vs. African Americans, χ^2 ^(p-value)
RS2814778 **A**/G	1	157441307	0.93	0.9	0.2	352.5 (0.000003)

RS6003 **A**/G	1	195297644	0.92	0.9	0.4	195.6 (0.000002)

RS2065160 C/**T**	1	203057600	0.91	0.9	0.6	85 (0.000001)

RS2752 G/**T**	1	232580494	0.92	0.5	0.8	68.3 (0.000001)

RS3287 **A**/G	2	54661161	0.93	0.7	0.4	64.5 (0.000001)

RS1824347 A/**G^Ψ^**	4	174001152	0.85	0.5	0.9	120.1 (0.000001)

RS3309 **A**/T	5	56128536	0.95	0.7	0.6	7.9 (0.005)

RS3317 A/**G**	5	112240050	0.93	0.5	0.8	69.5 (0.000001)

RS877826 **A**/C^**Ψ**^	5	138646696	0.83	0.3	0.8	156.9 (0.000002)

RS3340 **A**/G	5	153812060	0.91	0.8	0.9	13.4 (0.0002)

RS1928533 **C**/T^**Ψ**^	6	45617802	0.88	0.4	0.7	60.7 (0.000001)

RS1016461 C/**T^Ψ^**	6	69092970	0.85	0.5	0.8	63.2 (0.000001)

RS1538956 **G**/T^**Ψ**^	6	127005719	0.81	0.6	0.8	29.3 (0.00001)

RS2763 **C**/G	7	556186	0.90	0.9	0.8	13.6 (0.0002)

RS2161 A/**G**	7	97930442	0.94	0.7	0.5	29.9 (0.00001)

RS2740574 **A**/G	7	99220032	0.92	0.9	0.4	194.7 (0.000001)

RS285 C/**T**	8	19859469	0.89	0.5	0.8	66.0 (0.000001)

RS1888952 C/**T^Ψ^**	9	16248118	0.83	0.5	0.8	62.0 (0.000001)

RS594689 A/**G**	11	65392135	0.93	0.5	0.8	69.1 (0.000001)

RS1042602 A/**C**	11	88551344	0.90	0.6	0.9	80.0 (0.000001)

RS1800498 **C**/T	11	112796798	0.94	0.4	0.7	64.7 (0.000001)

RS1079598 C/**T**	11	112801484	0.86	0.7	0.8	8.7 (0.003)

RS5443 C/**T**	12	6825136	0.93	0.3	0.7	112.6 (0.000001)

RS898271 **A**/G^**Ψ**^	13	90539922	0.81	0.5	0.7	25.2 (0.000001)

RS1800404 **A**/G	15	25909368	0.93	0.7	0.3	113.5 (0.000001)

RS2891 **A**/G	17	3652275	0.93	0.5	0.8	69.0 (0.000001)

* Major allele in total combined population bolded.

^Ψ ^Indicates genotyped using TaqMan.

### Analyses

Model-based clustering for inferring population structure was performed using *Structure *software [3]. An admixture ancestry model was chosen to estimate the likelihood that the observed genotypic data corresponded to K = 1 to 5 underlying subpopulations. Per standard *Structure *procedures, missing genotypes were still inputted. The "burn-in period" and the number of Markov Chain Monte Carlo repetitions after "burn-in" were each chosen to be 10,000. Summary statistics converged for these values. For each K, the estimated Ln of the probability of K clusters (log Pr (X | K)) was generated. Similar self-reported ethnicity-specific analyses were also performed.

The ANCESTDIST (Boolean) function of *Structure *was implemented to assess information about the distribution of Q, the estimated membership coefficients for each individual in each cluster. When this function is activated, the output file includes the left- and right-hand ends of the probability intervals for each q(i). (A probability interval is the Bayesian analog of a confidence interval.)

## Findings

Demographic and risk factor characteristics by case-control status are described in Table 1. The mean age of the cases was 39.5 years and the mean age of control subjects was 37.8 years. Among cases, 51.5% were African American and among controls, 42.6% were African American. Cases were significantly more likely than controls to have a history of hypertension (p < .0001), diabetes (p < .0001), angina-MI (p < .0001), and to currently smoke cigarettes (p < .0001).

Table 2 lists the SNPs by chromosomal location, including genotype call rates, ethnicity-specific major allele frequencies and resultant χ^2 ^comparison values.

Table 3 details *Structure *output (log Pr (X | K) (denoted in Table 3 as Ln Prob) and Dirichlet parameter (α)) estimating the number of subpopulations (K) in our sample, K = 1 to 5. Results for the combined and ethnicity-specific analyses are presented. For the combined population, two subpopulations are likely because:

**Table 3:** Structure inference algorithm output (log Pr (X | K)) (denoted: Ln Prob) with Dirichlet parameter (α) estimating the number of populations (K) in our sample, K = 1 to 5.

	Combined Population		Caucasians		African Americans	
	**Ln Prob**	**alpha**	**Ln Prob**	**alpha**	**Ln Prob**	**alpha**

K = 1	-22523.9	n/a	-11068.1	n/a	-9169.1	n/a

K = 2	-20016.5	0.1541	-10738.3	0.0452	-8996.2	0.4928

K = 3	-19714.9	0.1059	-10671.9	0.0427	-9114.7	0.4271

K = 4	-19557.2	0.0481	-10585.0	0.0414	-9162.3	0.0957

K = 5	-19543.9	0.0482	-10633.8	0.0433	-9051.7	4.9466

1) log Pr (X | K) plateaus at K = 2.

2) Dirichlet parameter for amount of admixture (α) converges to a value < 0.2 once the Markov chain converges.

3) Most individuals are strongly assigned to one of the two populations.

**Figure 1:**
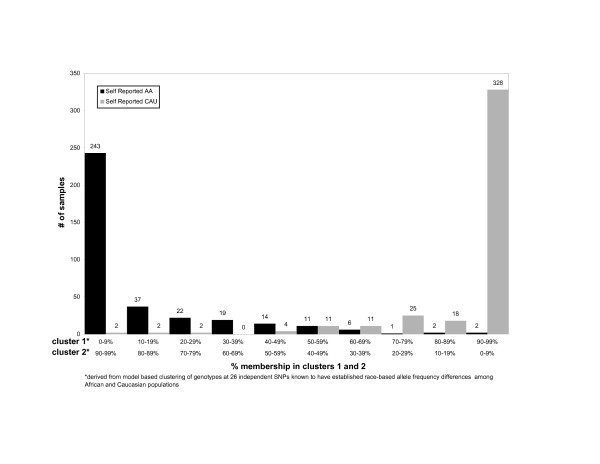
**Number of self reported African Americans and Caucasians as a function of percent membership in clusters 1 and 2**.

Figure 1 graphically demonstrates for K = 2 clusters, the estimated probability of self-reported Caucasians and African-Americans belonging to each cluster. Summarizing, 98% of self-reported Caucasians had an estimated probability ≥50% of belonging to cluster 1, while 94% of self-reported African-Americans had an estimated probability ≥50% of belonging to cluster 2. Further, 81% of self-reported Caucasians and 68% of self-reported African-Americans had an estimated probability ≥90% of belonging to clusters 1 and 2 respectively.

The *Structure *ANCESTDIST option provided the 90% probability intervals for each individual. Of the 760 individuals, 130 (17%) have overlapping probability intervals. Hence, 83% of the study population demonstrated individual ancestry proportion estimates that had non-overlapping 90% probability intervals.

Ethnicity specific exploratory analyses (demonstrated in Table 3) indicate some further substructure may be present among the self-reported Caucasians as log Pr (X | K) plateaus at K = 2 and α converges to a value < 0.2. When K = 2 among Caucasians alone, individuals distribute unevenly between the two clusters with 40% belonging to one cluster and 60% belonging to the other (data not shown). No further substructure was identified in our population of self-reported African-Americans as log Pr (X | K) does not plateau for K = 1 to 5 and α diverges.

## Discussion

Our results indicate that among the combined sample of African-American and Caucasian participants, self-reported ethnicity can serve as a proxy for genetic ancestry or relatedness. Furthermore, no large unknown subpopulation was identified. The ethnicity-specific analyses demonstrate no clear substructure in self-reported African American participants. This differs from the accepted idea that greater genetic diversity, as measured by linkage disequilibrium, is seen in populations of African origin. The lack of substructure in our African-American participants may be related to limitations of our panel. Interestingly, the ethnicity-specific analyses do demonstrate that some population substructure may exist among self-reported Caucasian participants. Evaluation of substructure in Americans of European decent has shown a course separation of European populations along a northeast to southwest axis [8]. In this light, our heterogeneous urban-based Caucasian population may partially explain the substructure present in our Caucasian participants. Notably, there are plans for the SPYW population to be part of a genome wide association study (GWAS) for ischemic stroke, thereby providing many more SNPs to better characterize the substructure of both the Caucasian and African-American participants. Another limitation of our study was the relatively low call rates, most notable for SNPs genotyped via the TaqMan platform. However, this should not have influenced our results because call rates did not differ significantly between cases and controls or those of self-reported African Americans and Caucasians (data not shown).

In summary, among the combined population, a small number of individuals were genetically more consistent with the other ancestry. Specifically, with a 50% ancestry threshold, 22 self-reported African-Americans were more consistent with Caucasian ancestry, while 10 self-reported Caucasians were more consistent with African-American ancestry. This information may be incorporated into future association analyses in various ways. Individuals not satisfying an ethnicity-based ancestry threshold could simply be removed from the study. Alternatively, as mentioned above, more null markers could be genotyped to improve the ancestry classification. Lastly, a variable incorporating percentage of ancestry could be introduced into the association analyses.

## Conclusion

Among our combined sample of African-American and Caucasian participants there is no large unknown subpopulation and self-reported ethnicity can serve as a proxy for genetic ancestry or relatedness. Ethnicity-specific analyses indicate that population substructure may exist among the Caucasian participants indicating that further studies are warranted.

## Competing interests

The authors declare that they have no competing interests.

## Authors' contributions

All authors certify that they participated in the conceptual design of this work, the analysis of the data, and the writing of the manuscript to take public responsibility for it. All authors reviewed the final version of the manuscript and approve it for publication. JBM, JWC, BDM and SJK: participated in the writing of the initial draft. JWC, MAW, BJS and SJK: participated in data collection. JBM, TDH and OCS: participated in the genotyping. JBM, JWC, JRO, LRM, BDM, and JDS: participated in the data analysis. All authors provided critiques of the final manuscript.
